# Abnormal Error Monitoring in Math-Anxious Individuals: Evidence from Error-Related Brain Potentials

**DOI:** 10.1371/journal.pone.0081143

**Published:** 2013-11-13

**Authors:** Macarena Suárez-Pellicioni, María Isabel Núñez-Peña, Àngels Colomé

**Affiliations:** 1 Department of Behavioural Sciences Methods, University of Barcelona, Barcelona, Spain; 2 Institute for Brain, Cognition and Behaviour, University of Barcelona, Barcelona, Spain; 3 Department of Basic Psychology, University of Barcelona, Barcelona, Spain; University of Gent, Belgium

## Abstract

This study used event-related brain potentials to investigate whether math anxiety is related to abnormal error monitoring processing. Seventeen high math-anxious (HMA) and seventeen low math-anxious (LMA) individuals were presented with a numerical and a classical Stroop task. Groups did not differ in terms of trait or state anxiety. We found enhanced error-related negativity (ERN) in the HMA group when subjects committed an error on the numerical Stroop task, but not on the classical Stroop task. Groups did not differ in terms of the correct-related negativity component (CRN), the error positivity component (Pe), classical behavioral measures or post-error measures. The amplitude of the ERN was negatively related to participants’ math anxiety scores, showing a more negative amplitude as the score increased. Moreover, using standardized low resolution electromagnetic tomography (sLORETA) we found greater activation of the insula in errors on a numerical task as compared to errors in a non-numerical task only for the HMA group. The results were interpreted according to the motivational significance theory of the ERN.

## Introduction

Math anxiety has been defined as a feeling of tension, apprehension or even dread, ranging from mild discomfort to extreme avoidance [1], which interferes with the ordinary manipulation of numbers and the solving of math problems [2]. The 2003 Program for International Student Assessment (PISA) report showed that more than 50% of 15-year-old students had feelings of insecurity and emotional stress when they were asked to solve mathematical problems. Similarly, behavioral studies have shown that math anxiety has a negative effect on a wide range of numerical and mathematical tasks, ranging from simple tasks like counting objects [3] to more complex arithmetical problems involving carrying [4]. Feelings of this kind make high math anxious individuals avoid situations that are math-intensive, and thus, to avoid educational tracks and career paths that depend on this discipline. Given the negative impact of math anxiety on mathematical learning and professional development, its study has emerged as a topic deserving intensive investigation. However, despite the increasing number of studies on math anxiety, error monitoring processing in this type of anxiety has not been assessed. The ability to learn from mistakes and to use that knowledge to guide future behavior is a critical cognitive skill, given that in many situations people rely upon internal self-monitoring to determine when their behavior is adequate or when adjustments need to be made. Studying how math anxious individuals perceive their self-generated errors, how they respond or adjust to them and how they perceive a numerical error as compared to a non-numerical one constitutes a very rich source of information that can improve our understanding of their difficulties with the subject and may identify a possible factor influencing the development and persistence of math anxiety.

A marker of performance monitoring that can be observed in brain activity is a very early negative component called error-related negativity (ERN) [5] or *error negativity* (Ne) [6]. The ERN is a response-locked event-related brain potential (ERP), observed as a sharp negative deflection at fronto-central recording sites along the midline (FCz or Fz electrode position) approximately 50-150 ms after an error is committed [5,7]. A wealth of data suggests that the ERN is generated in the Anterior cingulate cortex (ACC), a region of the medial prefrontal cortex that is richly interconnected with both limbic and frontal regions of the brain [6,8–11]. The precise cognitive mechanisms that generate the ERN are under debate, but the principal theories explaining its functional significance suggest that it reflects the detection of a mismatch between the representations of the actual and intended responses (*Mismatch theory*) [6], conflict monitoring in the ACC arising from multiple simultaneously active response tendencies (*Conflict monitoring theory*) [12] or the disinhibition of the ACC by dopamine neurons, which signal events as worse than anticipated (*Reinforcement and learning-based theories*) [13]. The principal shortcoming of these theories is that they do not account for motivational and individual differences. This limitation is overcome by the *motivational significance theory*, which suggests that the ERN may reflect error detection that is utilized for motivational ends; in this case, the amplitude of the ERN might be related to the significance of an error. For example, the ERN is enhanced when accuracy is emphasized over speed [5], when errors are associated with a high monetary risk or when errors are committed during social evaluation [14]. These and other studies have shown that more significant errors result in a larger ERN. Furthermore, this theory is often discussed in terms of affective processes. According to this interpretation, the ERN may be influenced by an individual’s emotional reaction to an error [15]. The idea is that affective evaluation occurs during error detection and that this evaluation varies along a continuum related to the distress caused by the commission of the error [16]. In fact, individuals with certain personality traits, characterized by increased sensitivity to errors, produce increased ERNs [17]. For example, enhanced ERN was found in patients with obsessive-compulsive disorder (OCD) [18,19], in undergraduate students with high obsessive compulsive characteristics [20], in patients with generalized anxiety disorder (GAD) [21], in undergraduates with high scores on measures of general anxiety and worry [22] and in participants scoring high on negative affect [23,24]. 

The counterpart of the ERN in correct trials is called correct-response negativity (CRN) [25]. The CRN is a small ERN-like component with the same temporal characteristics and scalp topography as the ERN. While its precise functional significance remains unclear, it may reflect a response comparison process [15], uncertainty about a correct response [25] or coactivation of correct and error responses [24]. The association between anxiety and this component is not yet clear. While several studies have found an enhancement in the ERN with higher levels of anxiety but no similar effect on the CRN [18,19], others have found an enhancement in both the ERN and CRN components [20,22,23,26–28]. While the first group of authors attribute their results to abnormal error monitoring (enhanced vigilance specifically for errors), the second group propose the enhancement of the two components as a sign of abnormal response monitoring in general. Some studies aiming to localize the source of this component have found that the CRN and the ERN represent the activity of the same underlying neuronal network [29,30] and thus, ostensibly reflect the same process. Nevertheless, other studies have found that the neural generators of the CRN were different and involved more posterior cingulate regions [31]. 

Error-related positivity (Pe) [32] appears after the ERN. This is a positive-going deflection in the waveform that is present between 200 and 400 ms after an error is committed, which exhibits a more posterior and central scalp distribution (maximum at Cz) than the ERN. It is also present after correct trials, but with a considerably attenuated amplitude [21,26,31]. The functional significance of the Pe is not as well understood as the ERN, but it has been principally associated with error awareness [33]. It has been shown that when errors are not recognized, the ERN amplitude and ACC activity remain unchanged, but the Pe amplitude is significantly lower than in recognized errors [34]. There are very few studies relating this component with anxiety and, generally, no significant differences have been found [19,21,35]. Nevertheless, other studies have suggested smaller Pe in high-anxious individuals [18,20]. The putative generator of Pe has also been estimated within the ACC region, the cingulate gyrus [36], and more posterior cingulate regions [37]. The exact distinction between ERN and Pe remains to be clarified in terms of both functional significance and anatomical sources. 

In the present study, we investigated differences in error monitoring as a function of math anxiety. Our objective was to help determine a possible factor in the development and maintenance of math anxiety and to further the understanding of the impairments experienced by the individuals who suffer from it. As far as we know, no study to date has investigated error-related brain potentials in high math-anxious individuals. To do so, we formed two groups with extreme levels of math anxiety, but who did not differ in terms of trait or state anxiety; consequently, group differences could not be attributed to general anxiety. Traditionally, error-related ERP components are elicited by having participants engage in a speeded response task. A good candidate is the Stroop task, given that participants have to deal quickly with contradictory information that makes them commit a sufficient number of errors. In our experiment participants performed a numerical Stroop task [38] (salient for the high math-anxious group) and a modified classical Stroop task (control task) [18]. We recorded the ongoing EEG and subsequently examined brain activity time-locked to both error responses (for the ERN) and correct responses (for the CRN). 

Based on the association between anxiety and internal error monitoring, enhanced ERN was expected in high math-anxious individuals only on the numerical Stroop task, which is the more salient task for this group. Moreover, given previous evidence of a significant negative correlation between the magnitude of the ERN enhancement and the severity of GAD patients [21], we expected to find the same significant negative correlation between the ERN amplitude and the self-reported level of math anxiety. We also analyzed whether math anxiety was associated only with erroneous responses, which would indicate abnormal error monitoring (enhanced vigilance specifically for errors), or with both error and correct trials (abnormal response monitoring in general). Furthermore, we analyzed later error-related components (Pe) as a function of math anxiety. As the bulk of evidence suggests that anxiety does not affect the Pe component, we expected to find no difference in this component between the high and low math anxious individuals for any task. Moreover, given the evidence from numerous anxiety-related studies [18,20,22,26], we expected to find no differences between the groups in terms of response time or error rates for any task. We also used standardized low resolution electromagnetic tomography (sLORETA) [39] to determine the brain electrical sources of the ERN, the CRN and the Pe components, in order to establish whether these error-related components are produced by the same or by slightly different neuroanatomical structures. Finally, differences in voxel activation between tasks in each group were analyzed. We expected that the HMA group would show a greater activation of emotional brain areas when committing an error in a numerical task as compared to an error in a non-numerical task. Obtaining this difference only for the HMA group, but not for the LMA one, might suggest that the fact of failing in a task involving numbers is perceived as an emotional negative event for individuals with high levels of math anxiety, a finding that would contribute to a better understanding of their avoidance of any situation involving the manipulation of numbers. 

## Methods

### Participants

Thirty-four healthy volunteers were tested in this study, half high math anxious and the other half low math-anxious. They were selected from a sample of 452 university students at the University of Barcelona who were assessed for math anxiety, trait anxiety and state anxiety [40,41] (see materials). These tests were administered only in the group formation phase and not during the experimental session. 

We initially tested 38 participants but four of them were not included in the analysis: two because of excessive artifacts and the other two because there was no low math-anxious counterpart. 

The low math-anxious group (henceforth, LMA) comprised seventeen participants who scored below the first quartile on the Shortened Mathematics Anxiety Rating Scale (sMARS) [40] while the high math-anxious group (henceforth, HMA) comprised seventeen participants who scored above the third quartile on the sMARS. 

Groups differed in math anxiety (*t*(32) = 19.37, *p* < .001), but not in trait anxiety (*t*(32) = .54, *p* = .59), state anxiety (*t*(32) = 1.42, *p* = .16), age (*t*(32) = .27, *p* = .78), years of formal education (*t*(32) = .74, *p* = .46), handedness (χ^2^ = 1.03, *p* = .31), ethnicity (χ^2^ = 0.0, *p* = 1) or gender distribution (χ^2^ = .18, *p* = .67). More detailed information about these variables is shown in Table 1. 

**Table 1:** Means and standard deviations (in brackets) for age, educational level, math anxiety, trait and state anxiety and frequencies for gender and manual dominance for the LMA and the HMA groups.

	Age	Gender	Manual Dominance	Educational level	sMARS	STAI-T	STAI-S
LMA	20.24 (2.07)	13	16	15.59 (1.87)	45.29 (5.19)	19.12 (10.43)	14.18 (8.20)
HMA	20.06 (1.60)	14	17	15.18 (1.28)	84.82 (6.61)	21.06 (10.43)	18.35 (8.90)

Note: LMA: low math-anxious; HMA: high math-anxious; Gender: number of women; Manual Dominance: number of right-handed; Educational level: number of years of formal education. sMARS: shortened Math Anxiety Rating Scale; STAI-T: Trait anxiety subscale from the STAI; STAI-S: State anxiety subscale from the STAI.

All participants had normal or corrected-to-normal visual acuity and did not report any history of neurological or psychiatric disorders. All were naïve as to the purposes of the study.

### Ethics Statement

Participants were paid for their participation and gave written informed consent before the experiment. The experimental protocol was approved by the Ethical Committee of the University of Barcelona and was in accordance with the Code of Ethics of the World Medical Association (Declaration of Helsinki).

## Materials

### Groups were formed according to the participants´ scores on the following tests

#### Shortened Mathematics Anxiety Rating Scale (sMARS)

This instrument measures anxiety by presenting 25 situations which may cause math anxiety grouped into three factors: math test anxiety, numerical task anxiety and math course anxiety. *Math test anxiety* (factor I) includes items reflecting apprehension about taking a test in mathematics or about receiving the results. Factor II, labeled *numerical task anxiety*, comprises items reflecting anxiety about executing numerical operations; Factor III, called *math course anxiety*, includes items related with enrolling on and attending a math course and some typical situations. In the present study, we used the Spanish version of the sMARS [40], whose scores have shown strong internal consistency (Cronbach’s alpha = .94) and high 7-week test-retest reliability (intra-class correlation coefficient = .72).

#### State-Trait Anxiety Inventory (STAI)

The STAI is a 40-item scale used to measure state (STAI-S) and trait (STAI-T) anxiety. Good to excellent internal consistency (Cronbach´s alpha = .86 - .95) and adequate 30-day test-retest reliability (State: *r* = .71-.76; Trait: *r* = .75-.86) have been reported for the Spanish version of this test [41]. 

Two tasks were presented to each participant during the recording session: a numerical Stroop task (salient for the HMA group) and a classical Stroop task (control task). 

#### The classical Stroop task

The classical Stroop task was the one proposed by Gehring et al., (2000), in which the words *ROJO* (red), *VERDE* (green) and *AZUL* (blue) were presented in either red or green ink on a computer monitor using a black background. Participants had to respond to the color of the ink in which the word was written, ignoring the color designated by the word. Stroop conditions could be congruent (the ink color matched the semantic meaning of the word, e.g. *ROJO* printed in red ink), incongruent (the color of the ink conflicted with the semantic meaning of the word, e.g. *ROJO* printed in green ink) or neutral (the word did not map directly to either response, e.g. *AZUL* printed in red ink) [18]. The words subtended view angles of 4.01°, 4.98° and 4.01° (horizontally) for *ROJO*, *VERDE* and *AZUL*, respectively, and 0.97° (vertically).

Participants were instructed to press the right or left mouse button in response to the color of the ink in which the word was written. Half of the participants were told to press the left button of the mouse when the color of the ink was red and the right button when the color of the ink was green, and the other half were told to do the opposite. Each trial began with a fixation sign (an asterisk) shown for 500 ms. After a 300 ms pause (a black screen), the word was shown for 200 ms and then followed by a 800 ms-black screen (maximum response windows of 1000 ms). A variable inter-trial interval (600-1100 ms) was used. Following the 24 trials of the training session, the participants received eight blocks of 42 trials (336 total trials). 

#### The numerical Stroop task

In the numerical Stroop task, the stimuli consisted of a pair of Arabic numbers shown simultaneously in the middle of the computer screen. There were four possible types of number pairs: 1-2, 1-8, 2-9, 8-9. Numbers were presented in three sizes: large (font size 80), neutral (font size 60) and small (font size 40). Stimulus pairs subtended view angles of 0.68°, 1.03° and 1.37° (horizontally) and 0.97°, 1.43° and 1.77° (vertically) for large, neutral and small size stimuli, respectively. The participants’ task consisted of responding to the number of higher numerical magnitude and ignoring the physical size. Number pairs were presented in three conditions: in the congruent condition, the number of larger numerical magnitude was also larger in physical size (e.g. 8 9), in the incongruent condition, the number of larger numerical magnitude was smaller in physical size (e.g. 8 9) and in the neutral condition the numbers only differed in numerical magnitude, but not in physical size (e.g. 8 9) [38]. Participants were instructed to indicate the number of larger numerical magnitude by pressing the left or right button of the mouse, depending on the side of the screen in which the number of larger magnitude had appeared. For instance, if the number that appeared on the left was larger in magnitude than the one on the right, participants were expected to press the left button of the mouse. The side on which the larger number appeared was counterbalanced, so there were two instances for all number pairs (e.g., 8 9 and 9 8). Each trial began with a fixation sign (an asterisk) shown for 500 ms. After a 300 ms pause (a black screen), a pair of numbers were shown for 300 ms and then followed by a 700 ms-black screen (maximum response windows of 1000 ms). Each trial was followed by a variable inter-trial interval ranging from 600 to 1100 ms. There were 10 blocks of 48 stimuli (480 total trials), preceded by 24 practice stimuli. 

**Figure 1:**
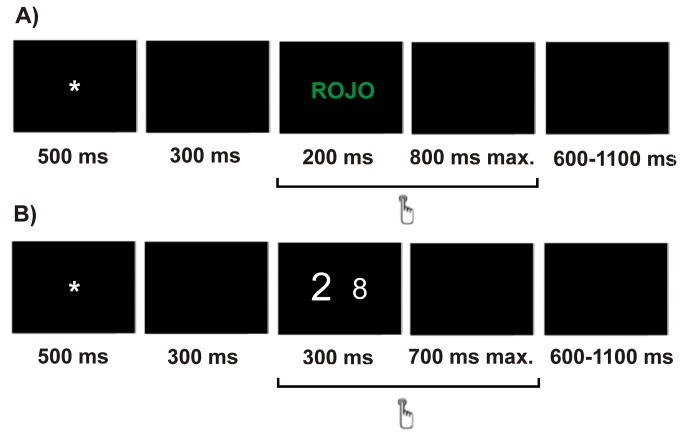
One trial structure of an incongruent stimulus of the classical (A) and numerical (B) Stroop tasks and its timing.

Figure 1 shows the sequential presentation of an incongruent stimulus and its timing for the classical (A) and the numerical (B) Stroop tasks. The order of the tasks was counterbalanced, so half of each group participated in the classical Stroop task first and then continued with the numerical task, while the other half did the same in reverse order. Within each task, the trials were randomly presented to each participant.

Both tasks included congruent, incongruent and neutral stimuli in equal proportions and all the stimuli were presented an equal number of times. Participants were asked to answer as fast and as accurately as possible. Moreover, in both tasks, a feedback message was displayed at the end of each block to facilitate error commission. The feedback was based on the participant’s performance on the block. If performance was 75% correct or lower, the message *Please try to be more accurate* was displayed; performance above 90% correct was followed by *Please try to respond faster*; otherwise, the message *You are doing a great job* was displayed. The feedback message was followed by a half minute rest. 

The E-prime 2.0 program (Psychology Software Tools Inc., Sharpsburg, PA, USA) was used to control the presentation and timing of the stimuli and the measurement of response accuracy and response times.

#### Procedure

Participants were tested individually. Upon entering the experimental room, they completed standard procedures concerning informed consent along with a demographics questionnaire asking their age, ethnicity, gender and number of years of formal education. Then, EEG/EOG sensor electrodes were attached and the participant was given detailed task instructions. Next, participants were seated 100 cm away from the computer screen in an electrically-shielded, sound-attenuating recording chamber. For each task, the experimental session began with a training period of 24 trials. When participants achieved 65% of hits in the training period, the recording session started. The training trials were only used to familiarize the participants with the task, so they were excluded from the statistical analysis. The experiment, including electrode placement and execution of the practice and test phases, lasted about 120 min. 

### Electrophysiological recording

The EEG was recorded with ANT hardware and software (B.V., Enschede, The Netherlands) from 64 electrodes positioned according to the extended 10/20 system, as well as two electrodes on the right and left mastoids, and mounted in a commercial WaveGuard EEG Cap (Eemagine Medical Imaging Solutions GmbH. ANT Advanced Neuro Technology). EEG channels were continuously digitized at a rate of 512 Hz by an ANT amplifier (B.V., Enschede, The Netherlands). A band-pass filter was set from 1.6 to 30 Hz, and electrode impedance was kept below 5 kΩ. The horizontal and vertical electrooculogram was recorded with electrodes placed at the outer canthus and below the right eye respectively. The common reference electrode was placed on the tip of the nose and ground was located at AFz. For figures, grand average waveforms were low-pass filtered at 15 Hz.

### Source localization

Source localization was carried out using standardized low-resolution brain electromagnetic tomography (sLORETA) [39] to identify the brain areas generating the ERN, CRN and Pe components. The brain activity of the two groups in each task and between the tasks in each group was also compared for the three components. sLORETA estimates the sources of activation on the basis of the standardized current density at each of 6239 voxels in the gray matter and the hippocampus of the MNI-reference brain with a spatial resolution of 5 mm. The calculation is based upon a linear weighted sum of the scalp electrical potentials, with the assumption that neighboring voxels have maximal similar electrical activity. sLORETA solutions are computed within a three-shell spherical model co-registered with the MNI152 digitized structural human brain atlas template [42]. Therefore, these solutions are given in three coordinates: *X* is the distance in millimeters to the right (+) or left (-) of midline, *y* is the distance anterior (+) or posterior (-) to the anterior commissure, and *z* is the distance above (+) or below (-) a horizontal plane through the anterior and posterior commissures. 

Under ideal conditions, solutions provided by sLORETA, which are based on distributed brain activity, have no localization bias and achieve reliable localization of possible underlying sources [43,44]. 

## Data Analysis

### Behavioral data

Medians of response time were calculated for each participant in each task. The response time (RT) for error and correct responses and the percentage of error responses were analyzed through analyses of variances (ANOVAs).

Firstly, an ANOVA was performed to analyze reaction time taking *Response type* (error and correct) and *Task* (numerical and classical) as within-subject factors and *Group* (LMA and HMA) as the between-subjects factor. 

Regarding the percentage of errors, an ANOVA was performed taking *Task* as within-subject factor and *Group* as the between subjects factor. 

In addition to reaction times and accuracy, we examined participants’ post-error measures. For post-error slowing we analyzed the median of reaction times following errors and following correct responses and carried out an ANOVA taking Previous *response type* (error and correct) and *Task* (numerical and classical) as within-subject factors and *Group* (LMA and HMA) as the between-subjects factor. Regarding post-error accuracy, we calculated the percentage of errors that followed error responses and the percentage of errors that followed correct responses. We carried out an ANOVA, taking Previous *response type* and *Task* as within-subject factors and *Group* as the between subjects factor.

The *F* value, the uncorrected degrees of freedom, the probability level following correction, the ε value (when appropriate) and the *η* ^2^ effect size index are given in the results section. 

### Error-related potentials

ERPs were averaged for each participant time-locked to the response onset, including error responses (for the ERN) and correct responses (for the CRN) for all experimental conditions. The averaged EEG epochs were rereferenced to the mastoids’ mean activity. The average was constructed from -400 to 600 ms epochs relative to the response onset. A 100-ms window prior to the response (-200 to -100 ms) served as the baseline. Trials with voltages exceeding ± 100 µV in any electrode were excluded from the ERP average. Ocular artifacts were identified and corrected with the eye-movement correction algorithm used in the EEprobe program (ANT, The Netherlands). Previous evidence suggests that the ERN component stabilizes using a minimum of six to eight error trials [45]. In our study, all participants had at least eight error trials in each task. Despite the ERN is typically quantified in the 0-100 ms window (e.g. [21]), in our study, both the ERN and the CRN components were quantified as a mean amplitude measure in the 50-90 ms window following a correct response (CRN) or an error response (ERN), given that this was the window where differences between groups were shown maximal.

A repeated measures ANOVA was performed on the ERP mean amplitude at Fz, taking *Response type* and *Task* as the within-subject factors and *Group* as the between-subjects factor. Another repeated measures ANOVA was performed on the ERP difference in amplitude (ERN-CRN) at Fz taking *Task* as the within-subject factor and *Group* as the between-subjects factor. 

Regarding the Pe component, we carried out a repeated measures ANOVA on the ERP mean amplitude in the 150-250 ms window at Cz, taking *Response type* and *Task* as within-subject factors and *Group* as the between-subjects factor. We performed tests of simple effects whenever an interaction was significant and used the Bonferroni correction to control for the increase in type I error. 

### Source analysis

The voxel-based sLORETA-images were calculated for each group in each task and were also compared between the two groups (LMA vs. HMA) and between the two tasks (classical and numerical) using the sLORETA-built-in voxelwise randomization tests (5000 permutations), based on statistical non-parametric mapping (SnPM; for details see 43). The differences in localization between groups and tasks were computed by a voxel-by-voxel *t*-test for independent measures of the average sLORETA-images over the 50-90 ms window for the ERN and CRN and over the 150-250 ms window for the Pe component. The statistical sLORETA analysis gives the exact significance thresholds, regardless of non-normality and corrected for multiple comparisons. The significant differences between conditions at respective MNI coordinates and Brodmann areas (BA) are reported in the results section. 

## Results

### Behavioral Data

Regarding reaction time, responses were faster on error trials (mean = 282.32, SEM = 5.41) than on correct trials (mean = 329.77, SEM = 5.29) (*F*(1,32) = 95.94, *p* < .001, *η* ^2^ = .75) and were slower on the numerical Stroop task (mean = 330.47, SEM = 5.58) compared with the classical Stroop one (mean = 281.63, SEM = 5.50) (*F*(1,32) = 75.20, *p* < .001, *η* ^2^ = .70). Moreover, the *Response type x Task* interaction was also significant (*F*(1,32) = 4.73, *p* = .03, *η* ^2^ = .12), showing greater differences between tasks when the participants committed an error (classical: mean = 254.08, SEM = 7.65; numerical: mean = 310.55, SEM = 5.13) than when they gave a correct response (classical: mean = 309.17, SEM = 5.35; numerical: mean = 350.38, SEM = 6.71) (*p* < .001). No group main effect or interaction reached statistical significance (all *p* values ≥ .23).

Concerning accuracy, more errors were committed on the numerical Stroop task (mean = 11.83, SEM = .94) than on the classical one (mean = 9.61, SEM = .85) (*F*(1,32) = 6.39, *p* = .01, *η* ^2^ = .16). No group main effect or interaction reached statistical significance (all *p* values ≥ .44). Accuracy and response time means and standard deviations are shown in Table 2.

**Table 2:** Means (of medians) and standard errors (in brackets) for behavioral and ERP measures.

	**Classical Stroop Task**		**Numerical Stroop Task**	
	**LMA**	**HMA**	**LMA**	**HMA**
**Response time (ms)**				
Error trials	251.88 (8.49)	256.29 (12.74)	305.76 (6.62)	315.35 (7.85)
Correct trials	309.76 (7.55)	308.59 (7.60)	339.88 (8.55)	360.88 (10.35)
**Accuracy**				
No. of error trials	32.94 (4.18)	30.06 (4.00)	59.65 (7.03)	54.00 (5.78)
No. of correct trials	288.24 (8.17)	301.00 (5.18)	420.35 (7.03)	426.00 (5.78)
% of error trials	10.24 (1.23)	8.99 (1.17)	12.42 (1.46)	11.25 (1.20)
**ERPs (µV)**				
ERN	-3.99 (.57)	-3.76 (.90)	-4.19 (.71)	-6.38 (.67)
CRN	1.68 (.81)	2.27 (.67)	-.79 (.61)	.13 (.44)
ERN-CRN	-5.67 (.92)	-6.03 (.77)	-3.39 (.52)	-5.67 (.92)
Pe on error trials	2.94 (.87)	1.69 (.84)	2.05 (.69)	.74 (.59)
Pe on correct trials	1.13 (.40)	.53 (.59)	1.41 (.39)	1.21 (.46)

Note: Voltage for ERN and CRN in Fz for the 50-90 ms time window; Voltage for Pe in Cz for the 150-250 ms time window.

#### Post-error measures

We found a significant main effect of *Previous response type* (*F*(1,32) = 38.36, *p* < .001, *η* ^2^ = .54), showing that participants were slower after committing an error (mean = 346.70, SEM = 7.27) than after correct responses (mean = 324.50, SEM = 5.41). We found a significant Task x Previous *response type* interaction (*F*(1,32) = 10.16, *p* = .003, *η* ^2^ = .24). This interaction was due to a difference between previous response types in each task, being greater for the classical task (RT after correct responses: mean = 303.38, SEM = 5.59; RT after error responses: mean = 335.00, SEM = 9.55) than for the numerical task (RT after correct responses: mean = 345.61, SEM = 6.74; RT after error responses: mean = 358.41, SEM = 7.49). No group effect or interaction reached statistical significance (all *p* values ≥ .13). 

Finally, with respect to post-error accuracy, the overall ANOVA showed a significant main effect of *Previous response type* (*F*(1,32) = 1720.55, *p* < .001, *η* ^2^ = .98), showing that there was a lower percentage of errors after errors (mean = 11.27, SEM = .93) than after correct responses (mean = 88.72, SEM = .93). All the other main effects and interactions were non-significant (all p values ≥ .11).

### Event-related potentials

#### Error-related negativity (ERN) *and* Correct-related negativity (CRN)

**Figure 2:**
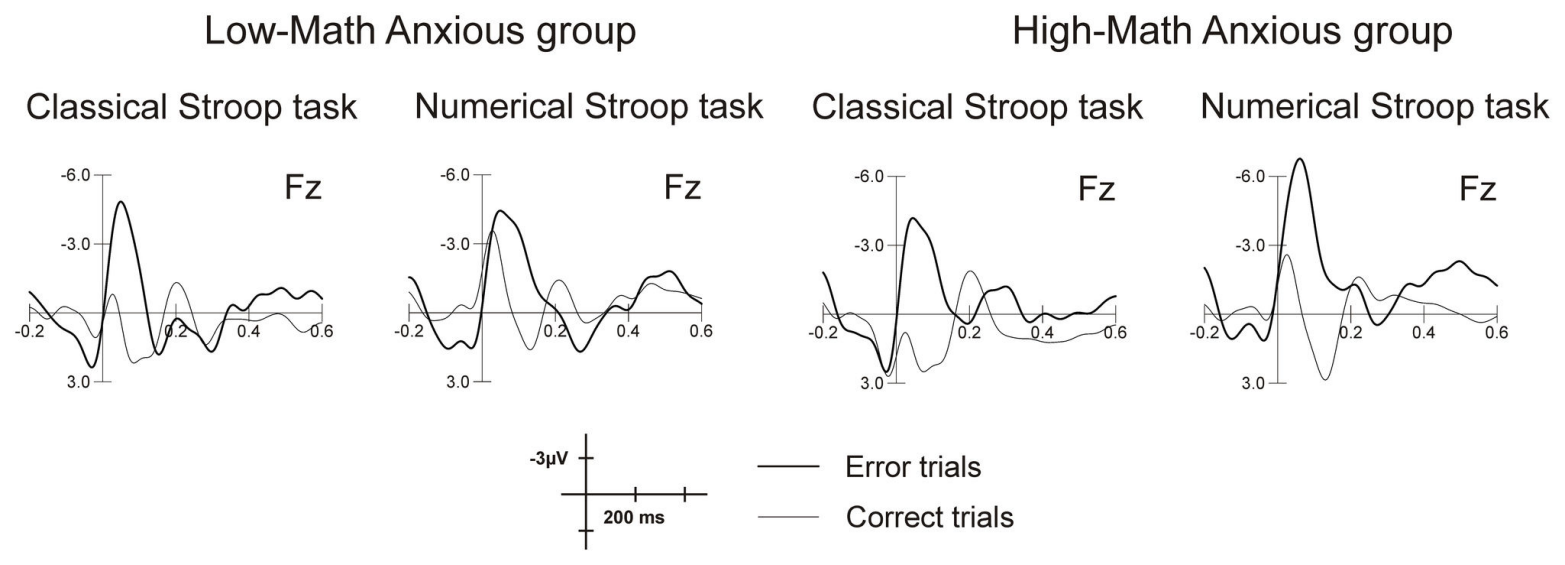
Raw grand average waves for correct and error trials for LMA and HMA groups in the classical and the numerical Stroop tasks at Fz.

Amplitude was more negative for error trials (mean = -4.58, SEM = .42) than for correct trials (mean = .82, SEM = .43) (*F*(1,32) = 157.41, *p < .001, η* ^2^ = .83). Figure 2 shows this early negativity for error commission as compared to correct responses in both the classical and the numerical Stroop tasks for the LMA and HMA groups at Fz. The mean amplitudes for the ERN and the CRN in the 50-90 ms windows are shown in Table 2. 

Moreover, the amplitude was more negative in the numerical (mean = -2.80, SEM = .37) than in the classical (mean = -.94, SEM = .43) Stroop task (*F*(1,32) = 30.48, *p* < .001*, η* ^2^ = .48). 

The Response type x Group interaction was also significant (*F*(1,32) = 2.07, *p* = .05*, η* ^2^ = .11), showing that, despite the fact that the two response types differed significantly in each group (*p* < .001), the HMA group showed a greater voltage difference between an error (mean = -5.07, SEM = .60) and a correct (mean = 1.20, SEM = .61) response than the LMA (error: mean = -.40, SEM = .60; correct: mean = .44, SEM = .61) one. Finally, the global ANOVA showed a significant *Group x Task x Response type* interaction (*F*(1,32) = 4.89, *p* = .03*, η* ^2^ = .13). No other main effect or interaction reached statistical significance (all p values ≥ .13)

**Figure 3:**
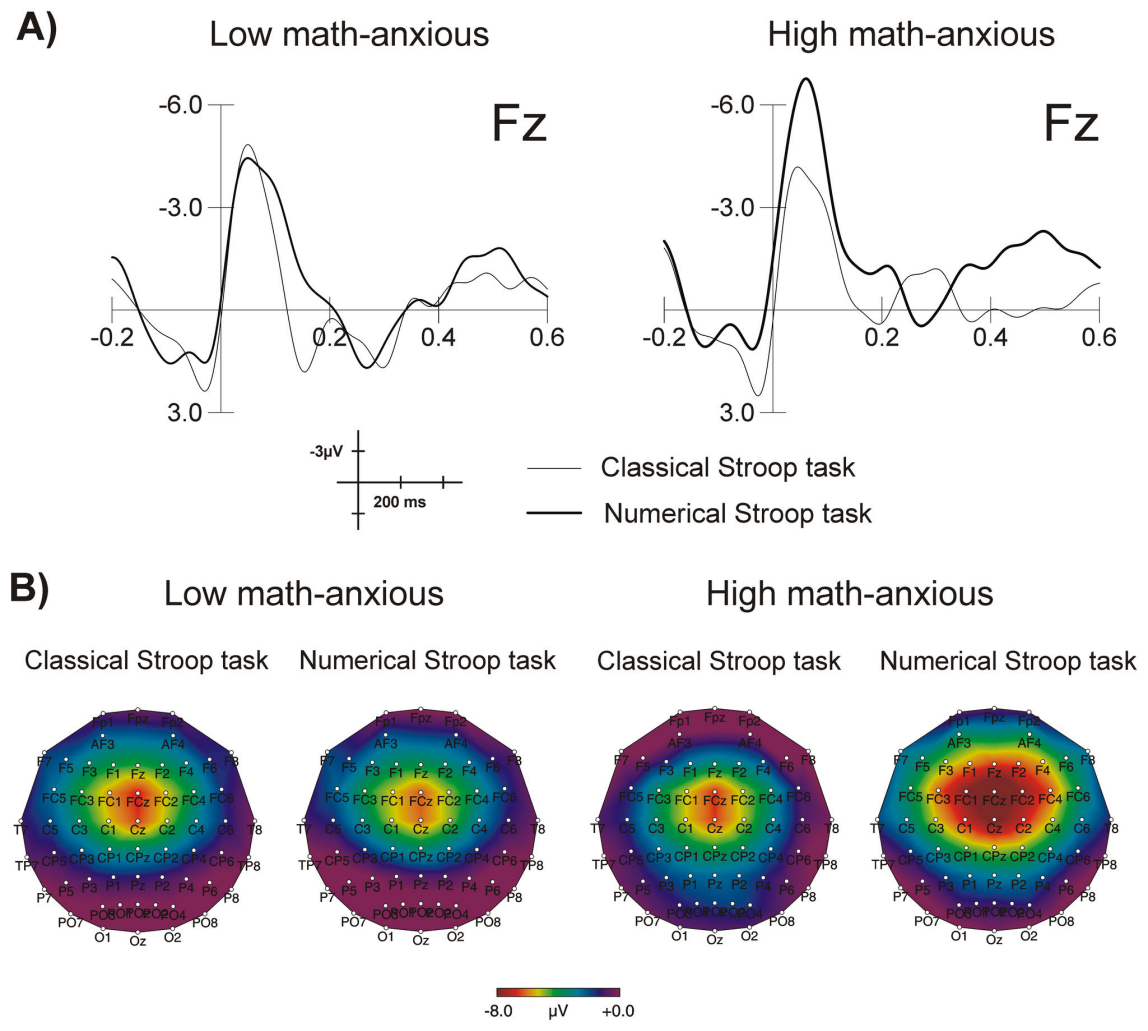
Image of error-related brain potentials. Grand average waveforms for the ERN at Fz for the LMA and the HMA groups in the numerical and the classical Stroop tasks (A) and scalp topography of the ERN component in the 50-90 ms window after the commission of an error for the LMA and the HMA groups in the classical and numerical Stroop tasks (B).

In order to analyze this *Group x Task x Response type* interaction further and to probe our hypothesis, we carried out a separate ANOVA for each group, comparing participants’ ERN in each task. While no significant effect of *Task* was shown for the LMA group (*F*(1,16) = .10, *p* = .75*, η* ^2^ = .006), this effect emerged for the HMA one (*F*(1,16) = 7.44, *p* = .01*, η* ^2^ = .31), the ERN being more negative for the numerical (mean = -6.38, SEM = .67) as compared to the classical (mean = -3.76, SEM = .90) Stroop task. Grand average waveforms elicited by errors for each group in each task at Fz are shown in Figure 3A. This figure shows a greater amplitude of the ERN for the HMA group when solving the numerical task as compared to the control task, while no difference between tasks can be appreciated for the LMA group. This difference is more evident in Figure 3B, where topographic maps for numerical and classical Stroop tasks are shown for both groups in the 50-90 ms window. Topographic maps were plotted using the EEProbe 3.1 program (ANT Software BV, Enschede, The Netherlands). 

#### ERP difference wave (ERN-CRN)

The analysis of the difference wave showed a significant *Task x Group* interaction (*F*(1,32) = 4.90, *p* = .03*, η* ^2^ = .13), showing that groups differ only on the numerical task (*F*(1,32) = 12.02, *p* = .002*, η* ^2^ = .27) but not on the classical (*F*(1,32) = .08, *p* = .76, *η* ^2^ = .003) task. The main effect of group also reached significance (*F*(1,32) =  4.08, *p* = .05, *η* ^2^ = .11), while the main effect of *Task* did not (*F*(1,32) =  2.06, *p* = .16, *η* ^2^ = .06). 

#### Error-related positivity (Pe)

**Figure 4:**
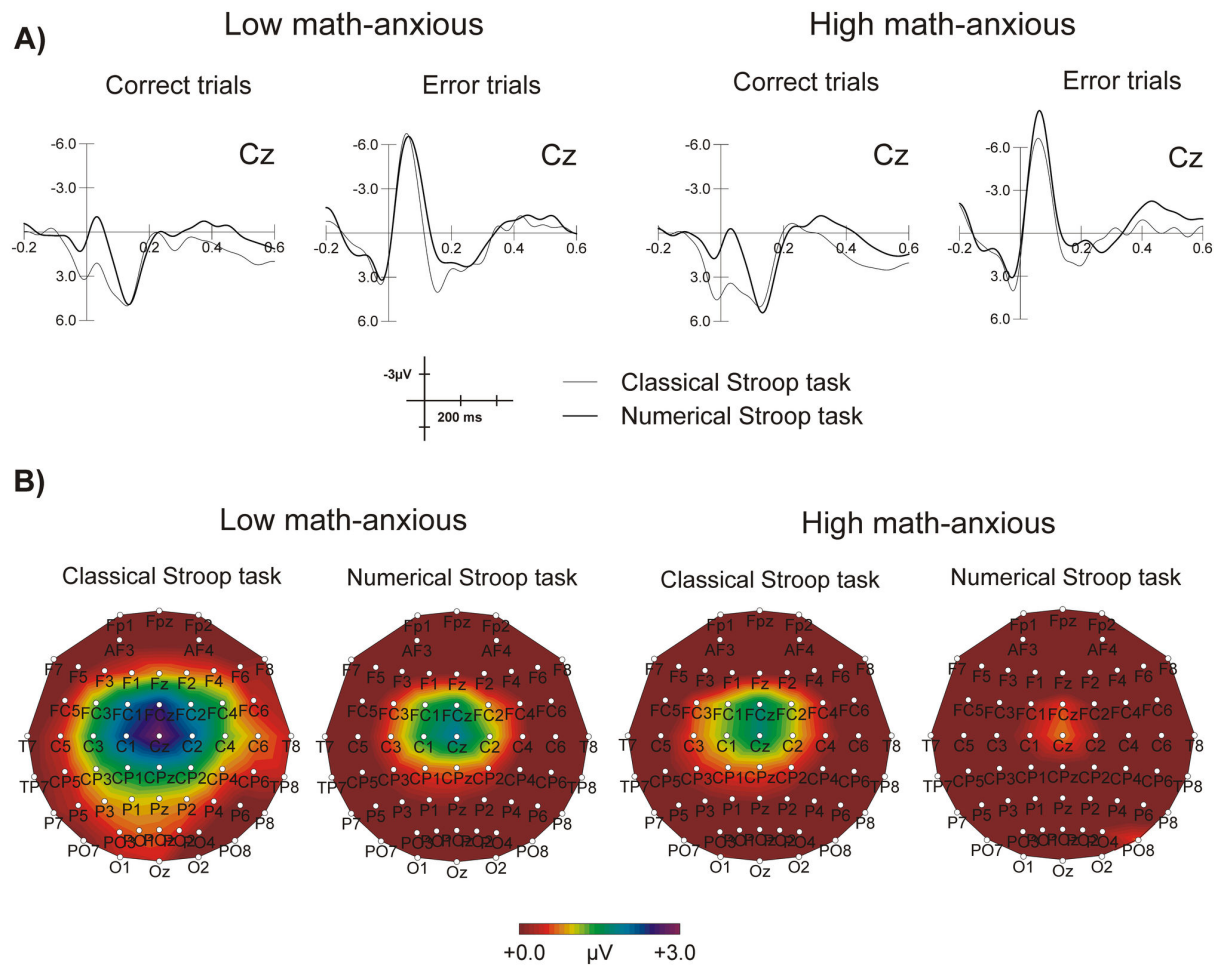
Image of error-related brain potentials. Grand average waveforms at Cz for the Pe component after errors and correct responses for the LMA and the HMA groups in the numerical and the classical Stroop tasks (A) and scalp topography of the Pe component after errors in the 150-250 ms window for the LMA and the HMA groups in the classical and numerical Stroop tasks (B).

Figure 4A shows grand average waveforms for the Pe component elicited by error and correct responses for each group in each task at Cz. The overall ANOVA showed a significant *Task x Response type* interaction (*F*(1,32) = 8.76, *p* = .006*, η* ^2^ = .21) showing that the response types differed only for the classical Stroop task (*t*(33) = 2.72*, p* = .01) but not for the numerical (*t*(33) = .22, *p* = .82); the amplitude was greater for an error (mean = 2.31, SEM = .60) than for a correct (mean = .83, SEM = .35) response. Besides the *Response type* main effect, which was marginally significant, all the *Group* main effect and interactions were far from significant (all *p* values ≥ .30). Table 2 also shows the mean amplitude for the Pe component in error and correct trials at Cz for the 150-250 ms window for both groups in the two tasks. Figure 4B shows the topographic maps for the Pe after errors in the numerical and the classical Stroop tasks for both groups in the 150-250 ms window. This figure shows that the Pe component after the commission of a numerical error seems to be reduced compared to the other conditions.

### Correlational analysis

We correlated participants’ scores on the sMARS test and in its three subscales with the mean electrophysiological activity for the ERN and CRN in the 50-90 ms window and for the Pe component in the 150-250 ms window for both tasks for the whole sample (n = 34). Pearson correlation coefficients and *p*-values are reported in Table 3. This table shows that as self-reported measures of math anxiety increased, the ERN for the numerical task became more negative. Nevertheless, this effect was absent for the classical task and for the CRN and Pe components in the numerical task. Moreover, it is worth mentioning that while the ERN-CRN difference wave correlated with the three subscales of the sMARS, the raw ERN wave correlated only with the Factor I subscale of this test, that is, with math test anxiety. 

**Table 3:** Pearson correlation coefficients (*p* values under brackets) between the sMARS scores and ERN, CRN and ERN-CRN components (at Fz) and Pe component (at Cz) amplitudes for the numerical and the classical Stroop tasks for the whole sample (n=34).

	Classical Stroop task				Numerical Stroop task			
	ERN	CRN	ERN-CRN	Pe	ERN	CRN	ERN-CRN	Pe
sMARS	.02 (.89)	-.09 (.60)	-.06 (.73)	-.15 (.37)	-.35 (.03)*	.17 (.32)	-.48 (.003)**	-.22 (.19)
Factor I	.02 (.87)	-.07 (.67)	-.04 (.81)	-.17 (.32)	-.38 (.02)*	.16 (.35)	-.50 (.002)**	-.22 (.19)
Factor II	.05 (.74)	-.13 (.43)	-.07 (.69)	-.07 (.67)	-.26 (.13)	.11 (.53)	-.34 (.04)*	-.20 (.24)
Factor III	-.01 (.94)	-.05 (.74)	-.06 (.72)	-.10 (.56)	-.19 (.25)	.19 (.26)	-.34 (.04)*	-.13 (.43)
STAI-T	.12 (.48)	.16 (.35)	-.03 (.85)	-.17 (.32)	-.01 (.91)	.14 (.41)	-.12 (.47)	-.30 (.07)
STAI-S	.14 (.39)	.09 (.60)	.05 (.77)	-.03 (.86)	-.04 (.81)	-.03 (.85)	-.01 (.92)	-.24 (.15)

Note. ** *p* < .01; * *p* < .05; Factor I: Math test anxiety; Factor II: Numerical task anxiety; Factor III: Math course anxiety; ERN: Error-related negativity; CRN: Correct-related negativity; Pe: error positivity (after errors).

### Source localization

#### For the ERN

Table 4 shows Brodmann areas of statistically stronger cerebral activation (*p* < .01) for the ERN (in red) for the LMA and the HMA groups in the classical and numerical Stroop tasks. This table shows that, as suggested by numerous studies, the ERN in all tasks and in the two groups implied, mainly, the activation of the Anterior cingulate cortex, the cingulate gyrus (both frontal and limbic), and the medial and middle frontal gyrus. 

**Table 4:** Brodmann areas of statistically stronger cerebral activation (***p* < **.01**) for the ERN (in bold), the CRN (in italics) and the Pe (without format) components for the LMA and the HMA groups in the classical and the numerical Stroop tasks**.

Lobe	Structure	Classical		Numerical	
		LMA	HMA	LMA	HMA
Limbic	Anterior Cingulate	**24, 32,** 33 / 32 / 24, 32, 33	**24, 32,** 33 / 24, 32	**24, 32,** 33 / 24	**10, 24, 25,** 32 / 24, 32
Frontal	Cingulate Gyrus	32 / 32	**32**	32 / 32	**6,** 32 / 32
Limbic	Cingulate Gyrus	**23, 24, 31,** 32 / 23 / 24, 31, 32	**24,** 32 / 24, 31, 32	**23, 24, 31,** 32 / 23*, 24,* 31/ 24, 31, 32	**23, 24, 31,** 32 / 23*, 24, 31*
Occipital	Cuneus	-	-	-	**30**
Sub-Lobar	Extra-Nuclear	*47*	*47*	*47*	*47*
Temporal	Fusiform Gyrus	*20, 36, 37*	*20, 36, 37*	20 / 20*, 36, 37*	*20, 36, 37*
Occipital	Fusiform Gyrus	-	*37*	*37*	*37*
Frontal	Inferior Frontal Gyrus	**6,** 9 / 13*, 45, 47*	*13, 45, 46, 47*	9 / 45*, 47*	**6, 9, 11,** 44 / 45*, 47*
Parietal	Inferior Parietal Lobule	40 / 40	*40*	40 / 40 / 40	40 / 40
Limbic	Inferior Temporal Gyrus	*20*	*20*	*20*	20 / 20
Temporal	Inferior Temporal Gyrus	*37*	-	*37*	*37*
Sub-lobar	Insula	13 / 13*, 45*	*13, 45*	13 / 13*, 45*	13 / 13*, 45*
Temporal	Insula	*41*	*41*	41 / 41	41 / 41
Occipital	Lingual Gyrus	-	-	*19*	**18,** 19 / 19
Frontal	Medial Frontal Gyrus	**6,** 32 / 9*, 10, 11* / 6, 8, 9	**6, 8, 9, 10,** 11 / 6, 9, 32	**9,** 32 / 6 / 6	**6, 8 , 9 , 10 , 11 ,** 32 / 6 / 6, 9
Frontal	Middle Frontal Gyrus	6 / 10*,* 46 / 6	**10,** 11 / 10*,* 46 / 6		**6, 9, 10,** 46 / 6
Temporal	Middle Temporal Gyrus	*21, 38*	*21, 38*	22 / 21*, 22, 37, 38, 39*	**20, 21, 22, 39** / *21, 22, 37, 38, 39*
Frontal	Orbital Gyrus	-	**11**	*47*	11 / 47
Frontal	Paracentral Lobule	**6,** 31 / 5	31	31 / 5	**5, 6,** 31 / 31
Limbic	Parahippocampal Gyrus	-	*19, 27, 28, 36, 37*	19 / 19*, 27, 28, 30, 34, 35, 36, 37*	**19, 27, 28, 30, 34, 35,** 36 / 19*, 27, 28, 30, 34, 35, 36, 37*
Frontal	Postcentral Gyrus	-	-	3 / 3	**3,** 4 / 3
Parietal	Postcentral Gyrus	*2,* 40 / 5	*2, 40, 43*	**2, 3,** 40 / 2*, 3,* 40 / 2, 3, 40	**1, 2, 3, 5, 40,** 43 / 2*, 3, 40*
Limbic	Posterior Cingulate	**23**	-	*23, 29, 30*	**23, 29, 30,** 31 / 23*, 29, 30*
Frontal	Precentral Gyrus	**6**	4	**4,** 6 / 4, 6	**4, 6, 43**
Frontal	Precuneus	-	-	31/ 31/ 31	31 / 31
Parietal	Precuneus	7 / 7	-	*7,* 31/ 7	**7,** 31 / 7*, 31*
Frontal	Rectal Gyrus	-	11 / 11	*11*	11/ 11
Frontal	Subcallosal Gyrus	-	-	*34*	*34*
Frontal	Sub-Gyral	6 / 10	6	-	**6, 8**
Limbic	Sub-Gyral	-	-	*19, 31*	31 / 19*, 31*
Parietal	Sub-Gyral	**2**	-	2 / 2*,* 40 / 2, 40	**2,** 40 / 2*, 40*
Temporal	Sub-Gyral	*37*	*21*	*13, 20, 21, 37*	*13, 20, 21, 37*
Frontal	Superior Frontal Gyrus	6 / 10*, 11*	**8, 10,** 11 / 6	**6**	**6, 8, 10, 11**
Parietal	Superior Parietal Lobule	-	-	5	**7**
Temporal	Superior Temporal Gyrus	*13, 22, 38, 41*	*13, 22, 38, 41, 42*	**13,** 41 / 13*, 22, 38, 41*	**13, 21, 22, 39, 41, 42** / *13, 22, 38, 41*
Parietal	Supramarginal Gyrus	-	-	-	40 / 40
Temporal	Transverse Temporal Gyrus	-	-	-	**41, 42**
Limbic	Uncus	*20, 28, 36, 38*	*20, 28, 36, 38*	*20, 34, 36, 38*	*20, 34, 36, 38*

**Figure 5:**
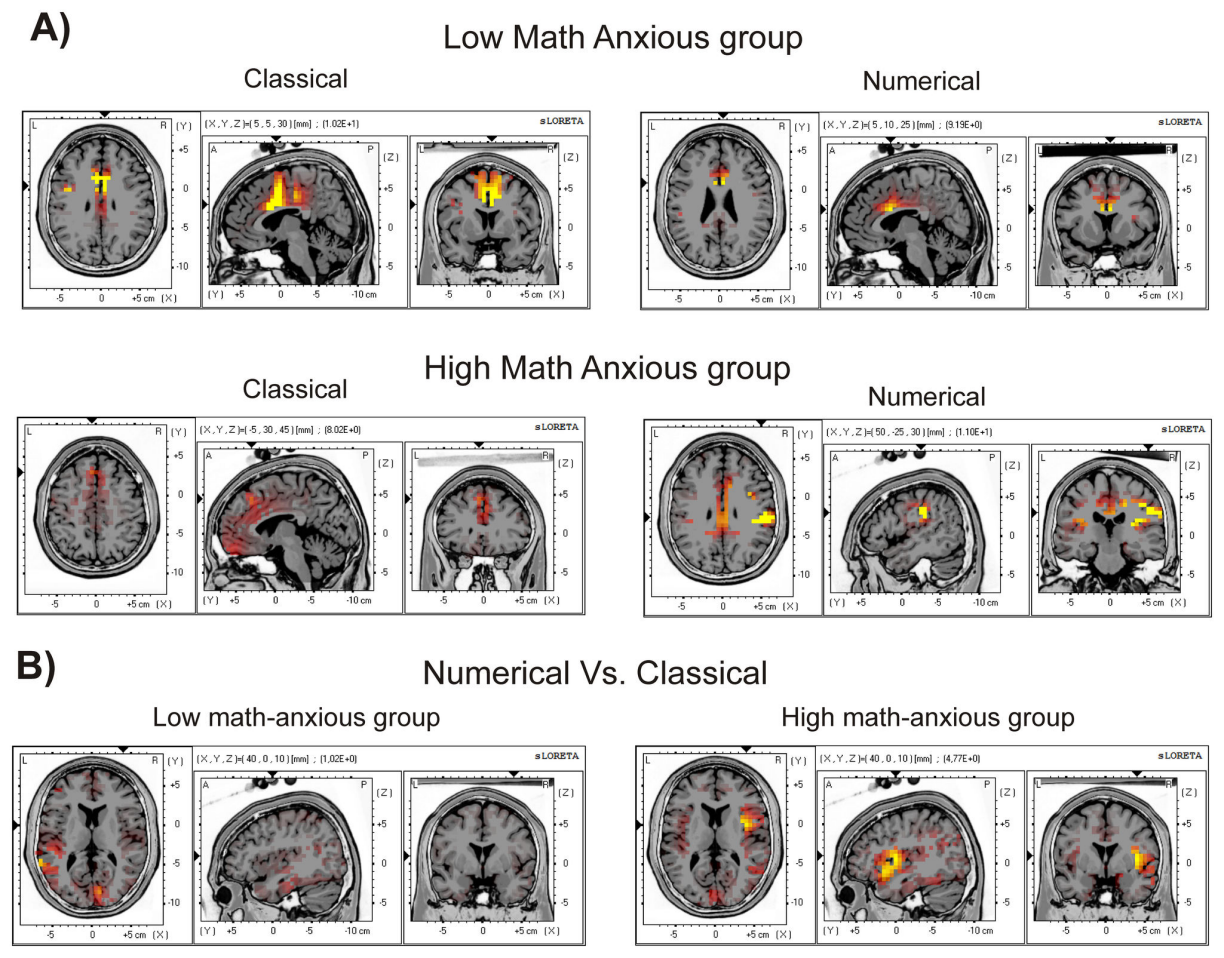
Images of neural activity computed with sLORETA. The images represent cortical areas showing significant activation (p < .01) for the ERN (50-90 ms window) for the LMA and the HMA group in the numerical and the classical Stroop task (A) and areas showing significant differences (p < .05) between the classical and the numerical Stroop tasks for the LMA and the HMA groups (B).

Very interestingly, sLORETA analysis showed that the HMA group activated different brain areas when committing an error on the numerical task compared with the classical task, and compared with the LMA group on any task. For example, the HMA group showed significant activation of the occipital cuneus, the superior parietal lobule, the transverse temporal gyrus and the supramarginal parietal gyrus. Moreover, within the brain structures that also showed activation for the other conditions, more Brodmann areas were activated in the case of errors committed by HMA individuals in the numerical task, for example, at the Anterior cingulate cortex (BA 10 and 25), inferior frontal gyrus (11 and 44), middle frontal gyrus (9 and 46), parietal post-central gyrus (1,5,43) or parahippocampal gyrus (27,28,30,34–36). Finally, voxel activation for the HMA group on the numerical task also involved areas that, for the LMA group (and the HMA in the classical task) were mainly active for correct responses (CRN), such as the inferior temporal gyrus (limbic), the middle temporal gyrus, the posterior cingulate, the limbic sub gyral or the parahippocampal gyrus (limbic). The involvement of greater voxel activation can be seen in Figure 5A, where cortical areas that showed significant activation (*p* < .01) for the ERN component (50-90 ms) for each group in each task are shown in red-to-yellow colors (*t*-values).

Despite the apparent activation of a greater number of voxels for the numerical errors in the HMA group, the statistical comparison between groups did not show significant results, either for the classical (*t* = 4.82, *p* = .67) nor for the numerical (*t* = 4.79, *p* = .69) Stroop task. 

Nevertheless, significant differences emerged when comparing tasks in each group. The results showed that while no differences were found for the LMA group (*t* = 4.89, *p* = .56), the HMA group showed a significant (*t* = 4.76, *p* = .04) difference in activation between tasks. Those differences were located at the insula, which showed a greater activation for the numerical task (max *t* value = 6.61) than for the classical task (max *t* value = 2.52). Table 5 shows the Brodmann areas, MNI coordinates and t values for the voxels presenting significant differences between tasks for the HMA group. Figure 5B shows, in red-to-yellow colors, cortical areas with significant (*p* < .05) differences between tasks for the LMA and the HMA groups, showing the greater difference between tasks for the HMA group. More concretely, when analyzing the HMA group’s brain activity in each task, a greater insular activation was found for the numerical (6.61) as compared to the classical (2.52) Stroop task at the right insula. 

**Table 5:** Areas of statistically higher localized brain activation for the numerical task compared with the classical task for the HMA group.

Lobe	Structure	B.A.	MNI coordinates (x,y,z)	*T*-value
Sub Lobar	Insula	13	40, 0, 5	4.83
Sub Lobar	Insula	13	40, 0, 10	4.77

Note: B.A: Brodmann Area; MNI: Montreal Neurological Institute; *t*-value of the statistical comparison with *p* < .05 for *t*-values above 4.76 (threshold).

#### For the CRN

Table 4 shows Brodmann areas with statistically stronger cerebral activation (*p* < .01) for the CRN (in blue) for the LMA and the HMA groups in the classical and the numerical Stroop tasks. This table shows that the CRN activated some areas previously found to activate the ERN, such as the cingulate gyrus (limbic lobe), inferior parietal lobule, the insula or the precuneus. Moreover, in other cases the CRN activated the same brain structures as the ERN, but different Brodmann areas were involved in the generation of each component. For example, in the case of the inferior frontal gyrus, while the activation of the ERN was located at Brodmann areas 6, 9, 11 and 44, the CRN activated different areas (13,45–47). Despite the overlapping of some areas in the generation of the ERN and the CRN, the CRN also showed a specific activation of certain brain regions such as the uncus, the temporal sub gyral, the extra-nuclear (sub lobar), the temporal and occipital fusiform gyrus or the inferior temporal gyrus (limbic and temporal). Very interestingly, the CRN showed no voxel activation at the anterior cingulated. 

The comparison between tasks did not reach significance either for the LMA group (*t* = 4.73, *p* = .44) or for the HMA group (*t* = 4.76, *p* = .29). Similarly, groups showed non-significant differences both for the classical (*t* = 4.64, *p* = .98) and for the numerical (*t* = 4.71, *p* = .86) tasks. 

#### For the ERN vs CRN

**Figure 6:**
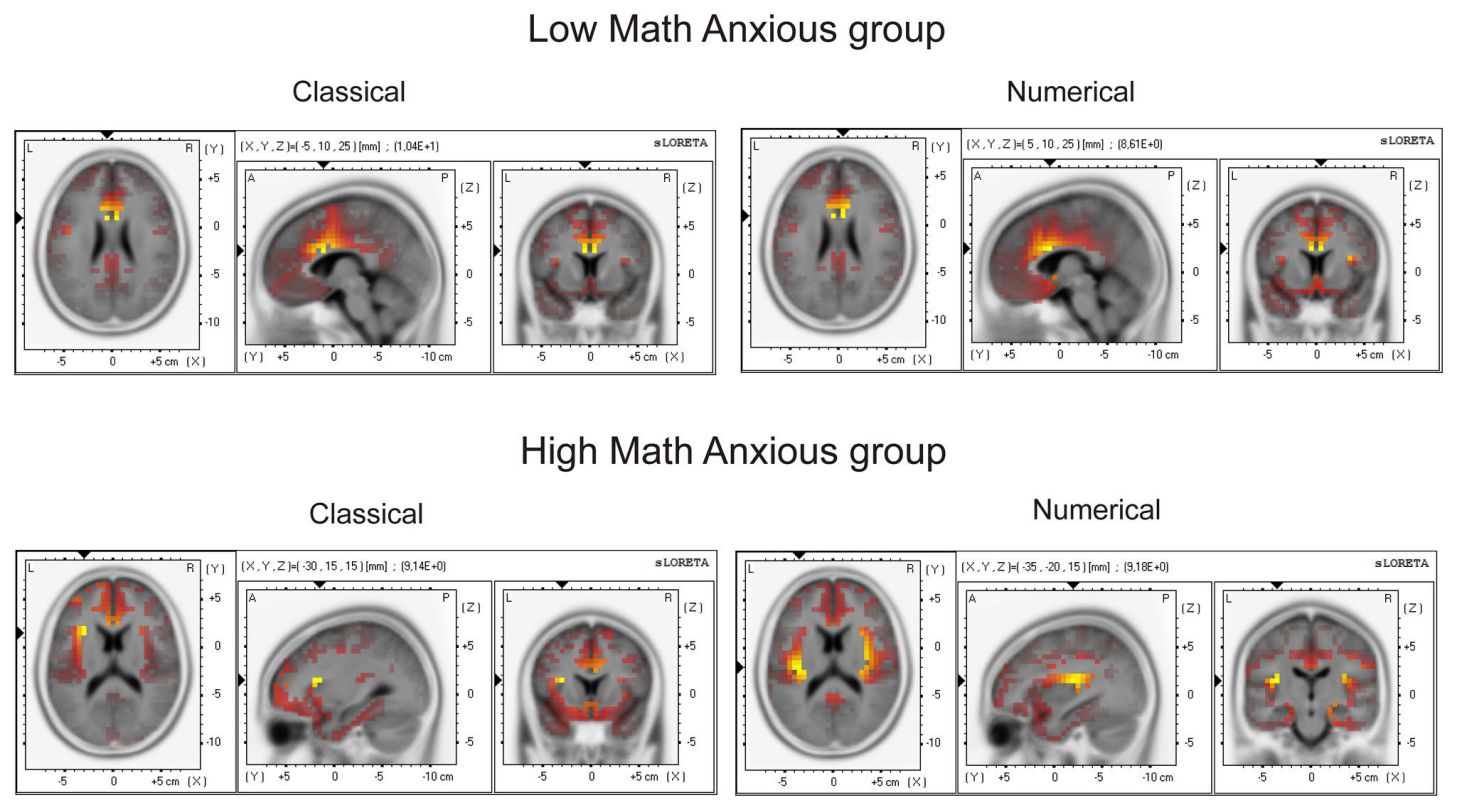
Images of neural activity computed with sLORETA. The images represent cortical areas showing significant activation (p < .01) for the ERN as compared to the CRN in the 50-90 ms window for the LMA and the HMA groups in the numerical and the classical Stroop tasks.

Figure 6 presents, in red to yellow colors (*t* values), the cortical areas that showed significant activation (*p* < .01) for the ERN as compared to the CRN, for the two tasks in the two groups. As expected, for all conditions, this differential activity was shown mainly in the Anterior cingulate cortex (Brodmann areas 24, 32, 33) and in the cingulated gyrus (Brodmann areas 24 and 33). Nevertheless, for the HMA group in the numerical condition, there was an area that showed even a greater activation, the insula (Brodmann area 13). 

#### For the Pe component

Table 4 shows Brodmann areas with statistically stronger cerebral activation (*p* < .01) for the Pe component (in green) for the LMA and the HMA groups in the classical and the numerical Stroop tasks. Like the ERN, the Pe showed significant voxel activation at the Anterior cingulate cortex, the frontal cingulate gyrus, the precuneus, the limbic cingulate gyrus (except for HMA in the numerical task), frontal paracentral lobule and the middle and medial frontal gyrus. Nevertheless, in contrast to the CRN, which showed activation of different areas with respect to ERN, the Pe component showed no activation of different brain structures, that is, all the activated brain areas also showed activation for the ERN. 

As in the case of the CRN, the comparison between tasks showed no statistical differences for the LMA (*t* = 4.78, *p* = .18) and the HMA (*t* = 4.60, *p* = .78) group. Neither did the differences between groups reach significance for the classical (*t* = 4.66, *p* = .42) or numerical (*t* = 4.64, *p* = .84) tasks. 

## Discussion

This study aimed to investigate error monitoring processing in individuals high in math anxiety in order to improve our understanding of the difficulties experienced by individuals with high math anxiety when they have to manipulate numbers. To the best of our knowledge, this is the first time that error monitoring processes have been explored in a cohort of this kind. To do so, we formed two groups that presented extreme scores of math anxiety, but did not differ in trait or state anxiety. This adds value to the study, as it enables us to rule out the possibility that any differences between the groups were due to general anxiety. Both groups had to solve a numerical Stroop task (more salient for the HMA group) and a classical Stroop task (control task). We used ERPs to analyze the electrophysiological response after errors and after correct responses. We expected to find differences between tasks only for the HMA group.

Our results confirmed our hypotheses. Consistent with studies of other anxiety disorders [18,20,22–24], non-clinical individuals with a high level of math anxiety were characterized by increased error-related brain activity and by a greater difference between the ERN and the CRN when they solved a numerical task, but not when they solved a task involving non-numerical stimuli. The specificity of these results for the ERN but not for the CRN suggests that HMA individuals differ from their LMA counterparts not in generic response monitoring processes, but specifically in the evaluation of errors [18,31].

With respect to the Pe component, neither the group main effect nor the interactions reached statistical significance, which corroborates the results of previous studies claiming that individual differences in anxiety do not seem to modulate later and more elaborate stages of error monitoring [19,21,35,46]. Nevertheless, we found a significant difference between response types, with the Pe component being more positive after errors than after correct responses (see 46,47 for similar results). However, this effect was only found for the classical Stroop task, while in the numerical task both errors and correct responses generated a very similar Pe component. This might be due to a reduction of the Pe component for the numerical errors in the HMA group (shown clearly in Figure 4B). In this respect, despite differences between groups were non-significant, this tendency of the HMA group to show smaller Pe amplitudes after errors has also been found previously [18,20]. Given that the Pe component is considered to show conscious error processing, this finding could be suggesting that HMA individuals might not be fully conscious about having committed a numerical error, as compared to an error in the non-numerical task. The relationship between the Pe component and math anxiety deserve further research, especially because of the great consequences that this possible lack of error consciousness could have in the process of mathematical learning. Regarding source localization analysis, the ERN activity mainly involved the Anterior cingulate cortex (Brodmann areas 24, 32 and 33) [9,11], and the medial and middle frontal gyrus (Brodmann area 6) [36], corresponding to the Supplemental motor area (SMA) (adjacent to the caudal part of the Anterior cingulate cortex) which has also been suggested to be a generator of the ERN [48]. The activation as well of some voxels at the insula, precuneus or posterior cingulate areas suggests a distributed error processing in the human system (see also 49). As for the CRN, it did not activate anterior cingulated brain areas. Hence, our results corroborate previous evidence suggesting that the CRN and the ERN involve different neural generators, with a greater involvement of posterior cingulate areas for the CRN and of anterior cingulated areas for the ERN [31]. The Pe showed activation mainly of the Anterior cingulate cortex (Brodmann area 24) and cingulate gyrus [36] and voxel activation seemed to be restricted to those areas that showed activation for the ERN, suggesting that these components were generated by the same ACC regions (see also 50,51). 

Moreover, across both groups, greater levels of math anxiety were associated with larger ERN only on the numerical task. A negative correlation between the level of anxiety and the amplitude of ERN has also been obtained in studies exploring GAD patients [21]. Interestingly, despite the fact that the ERN-CRN difference wave correlated with the three subscales of the sMARS, for the ERN raw wave the correlation only held for the Factor I subscale, that is, for math test anxiety. This could be suggesting that it is the evaluative aspect of math anxiety (math test anxiety) that best explains the relationship between math anxiety and error monitoring in our sample. In fact, previous studies have found that the presence of overt performance evaluation led to increased ERN responses compared to a non-evaluation condition [52]. 

Despite the differences found in electrophysiological measures, the groups did not differ in reaction time and percentage of errors [18,20,22–24,53]. This suggests that the exaggerated processing of errors in the HMA group (enhanced ERN) did not lead to increased behavioral regulation, which might imply an inefficient action monitoring. Regarding post-error measures, we found the classical slow-down in reaction time and increase in accuracy after error commission. Groups did not differ on these measures, in agreement with numerous previous reports of differences between groups on the ERN amplitude but not on the post-error slowing effect [21,26,27,54] and which suggests a preserved post-error adaptation effect in both groups. 

Although there were no differences between groups on behavioral measures, we found that the numerical Stroop task took more time and induced more errors than the classical one, suggesting perhaps that the numerical task was slightly more difficult. Consequently, it might be the case that amplitude differences in the groups’ ERN in this task were not only due to its numerical nature but also to its level of difficulty. Two findings argue against this interpretation. Firstly, behavioral measures showed that both groups needed approximately the same time to answer to the numerical task and committed approximately the same number of errors on it. Consequently, nothing suggests that the numerical task was more difficult for the HMA than for the LMA group. Secondly, previous research suggests that the increased difficulty of the task has no effect on the ERN or, if anything, it reduces ERN amplitudes. For example, Pailing and Segalowitz (2004) showed that tasks with higher error rates did not lead to a significantly larger or smaller ERN when compared to tasks with lower error rates [55]. According to this evidence, the fact that the numerical Stroop task was the one with higher error rates does not explain the larger ERN amplitude we found on it. However, a very recent study by Kaczkurkin (2013) showed that increasing task difficulty during a flanker task attenuated ERN amplitudes and enhanced CRN amplitudes, in direct contrast to our findings (enhanced ERN for the more difficult numerical task) [56]. Consequently, we consider that we can rule out the possibility that the ERN differences in amplitude in the numerical task could be attributed to task difficulty.

So, what does this enhanced ERN in the HMA group show? As we mentioned in the introduction, the functional significance of the ERN is still unclear. Two of the most important theories attempting to explain the meaning of the error-related negativity are the conflict monitoring theory and the reinforcement learning theory. Both theories contend that the variation of the magnitude of the ERN is predicted by behavioral measures, so the more frequent errors give rise to decreased ERN and the degree of post-error slowing is related to the magnitude of the ERN [57,58]. Contrarily to these claims, in our study, more frequent errors (errors in the numerical task) generated an increased ERN; the difference was unaccompanied by changes in behavioral or post-error measures, suggesting no relationship between this ERN component and current or subsequent behavior. For this reason, these theories seem unsuitable for explaining our results. 

However, the lack of differences in behavioral measures does not necessarily mean that both groups were dealing with the task identically: it might be that the HMA required greater effort in order to show a comparable level of performance in the numerical task to their LMA counterparts. This is exactly what the processing efficiency theory [59] and its extension, the attentional control theory [60], predict. According to these theories, anxiety influences *processing efficiency* (relationship between task performance and the amount of attentional resources spent on solving it) to a greater extent than *performance effectiveness* (quality of task performance). This effect is explained as a consequence of a deficient attentional control in anxious people, which would allow the attentional resources to be allocated to internal threatening stimuli (i.e., worrying thoughts) and consequently reducing the resources devoted to solving the task in hand. As a result, in order to compensate for this resource depletion, anxious people would increase their cognitive effort. Previous evidence has suggested that errors, being associated with cognitive as well as affective correlates, may reflect this effect of increased effort [31]. More specifically, the attentional control theory predicts that errors in high anxious individuals not only imply a *quantitative* difference between anxious groups (enhanced ERN for the high anxious group compared with the low anxious group), but also a *qualitative* difference, which would imply a different pattern of neural activity for the high anxious group, especially in areas involved in emotion processing and cognitive control. In this sense, while most studies have shown quantitative differences between anxious groups [17,18,21,61] others have also found very interesting qualitative differences [31]. For example, Aarts and Pourtois (2010) showed a different configuration of intracranial generators for the ERN between low and high-anxious individuals, with the involvement of more dorsal ACC regions for the low-anxious participants (Brodmann areas 24) and more anterior regions for high-anxious participants. In our study, although sLORETA images seem to suggest a trend for HMA individuals to activate more brain structures than their LMA counterparts when they commit a numerical error, strictly speaking differences in voxel activation between groups were not significant.

To conclude this review of the theories aiming to interpret the ERN component, many recent studies have suggested the idea that errors not only provide *cold* cognitive information, but also convey an important emotional significance and affective reaction [10,54,62]. This is the main idea of the motivational theory of the ERN. The bulk of evidence relating ERN and affect come from studies of anxiety. Several studies have reported an enhanced ERN amplitude for high anxious individuals compared with their low anxious peers [18,20,21,61], which is interpreted as reflecting high anxious individuals’ greater sensitivity and concern over errors [21,52]. Our study seems to extend those findings to math anxiety by showing an increased ERN for the HMA group compared to the LMA group for the task that was more salient for them. Furthermore, the sLORETA results showed significantly greater voxel activation at the right insula for the numerical task than for the classical task only for the HMA group. Previous research studying performance monitoring had already reported error-related signal increases at the insula [63–68]. The insula has been suggested to be of relevance for interoception, which can be defined as the sense of the physiological condition of the entire body [69–71]. In this respect, in a numerical version of the Stroop task, insular cortex activity was related to errors and sympathetic arousal measured via pupil diameter [72]. Classical theories of emotion posit that this awareness of one's internal bodily states is a key component of emotional experience. Several avenues of research suggest that the altered insular function is a feature of many anxiety disorders [73]. The right insula, in particular, has been associated with the extent of *interoceptive awareness* and discomfort with one's own physiological response (e.g. heart rate) to emotionally valent pictures [74] and anticipation of emotionally aversive stimuli has been shown to activate the right insular cortex [75]. Functional imaging research suggests that activity in the anterior insular cortex, particularly the right insula, may both mediate anxiety sensitivity and play a role in the pathophysiology of phobias. It is well known that errors are salient events that trigger a cascade of central nervous and autonomous changes such as skin conductance response [27], heart rate deceleration [22,23], pupil dilation [76], amygdala activity [62] and potentiated defensive startle reflexes [54]. As a consequence, it could be the case that the activation of the right insula found for the HMA group when committing a numerical error could be suggesting that this type of error could have generated a greater physiological response in HMA individuals, and that the identification of some of these subtle somatic symptoms might have increased their feeling of distress, showing the greater insular activation and the greater ERN amplitude. In this line, Lyons and Beilock, (2012), using functional magnetic resonance imaging (fMRI), reported an association between the insular cortex activation and subjective ratings of math anxiety when participants anticipated an upcoming math task [77]. Given that interoception has been shown to increase with heightened levels of anxiety and thus leads to increased sensitivity to physical pain, these authors interpreted this result as showing that even anticipating the unpleasant event of solving a math task was associated with the activation of neural regions involved in pain processing in HMA individuals. Despite this topic deserves further intensive research, our results could be suggesting that a HMA’s brain might perceive a numerical error as painful. 

It is worth mentioning that although we did not obtain insular cortex activation for the Pe component, this area has also been shown to be activated at this later stage of error processing [78] for conscious errors. In this case, it has been suggested that the spatio-temporal dynamics comprise a sequence of brain processes in the posterior cingulated (ERN), left insula (Pe component) and right orbito-frontal cortex (post-Pe) [79]. Nevertheless, the studies associating the conscious perception of errors and the insula have been designed to elicit conscious and unconscious errors, and the two types of errors have been analyzed separately. Since the main objective of this study was not to investigate error awareness, we did not distinguish between conscious and non-conscious errors, and this might be the reason why the insula did not show a significant activation for the Pe component in our study. 

To sum up, this is the first study showing abnormal error monitoring in individuals high in math anxiety. Our data suggest that HMA individuals seem to be hypersensitive to self-generated errors in numerical tasks, an effect shown by enhanced ERN and increased insular activation exclusively for numerical errors. Hence, our study also provides evidence that the ERN component conveys information beyond simple error detection and also reflects an affective evaluation of errors [16,17]. The fact that errors in numerical tasks are perceived as abnormally salient or aversive probably constitutes a contributory factor in the development and maintenance of math anxiety. These negative feelings may contribute to *global avoidance* [2,80], that is, the documented tendency of HMA individuals to avoid situations that are math intensive, such as the mathematics curriculum during formal education. This avoidance has an undesired effect on performance, given that it reduces their level of expertise in advanced mathematics. Given the importance of mathematics for academic and professional development and the poorer perspectives for those students suffering from math anxiety, this is a topic that deserves intensive investigation. 
