# Correction: Abnormal Error Monitoring in Math-Anxious Individuals: Evidence from Error-Related Brain Potentials

**DOI:** 10.1371/annotation/6f541903-ba9a-4b3f-b2d4-546f172b4477

**Published:** 2014-01-02

**Authors:** Macarena Suárez-Pellicioni, María Isabel Núñez-Peña, Àngels Colomé

The text formatting for Table 4 is incorrect and the caption is missing in the PDF version. Please see the corrected Table 4 here: 

**Figure:**
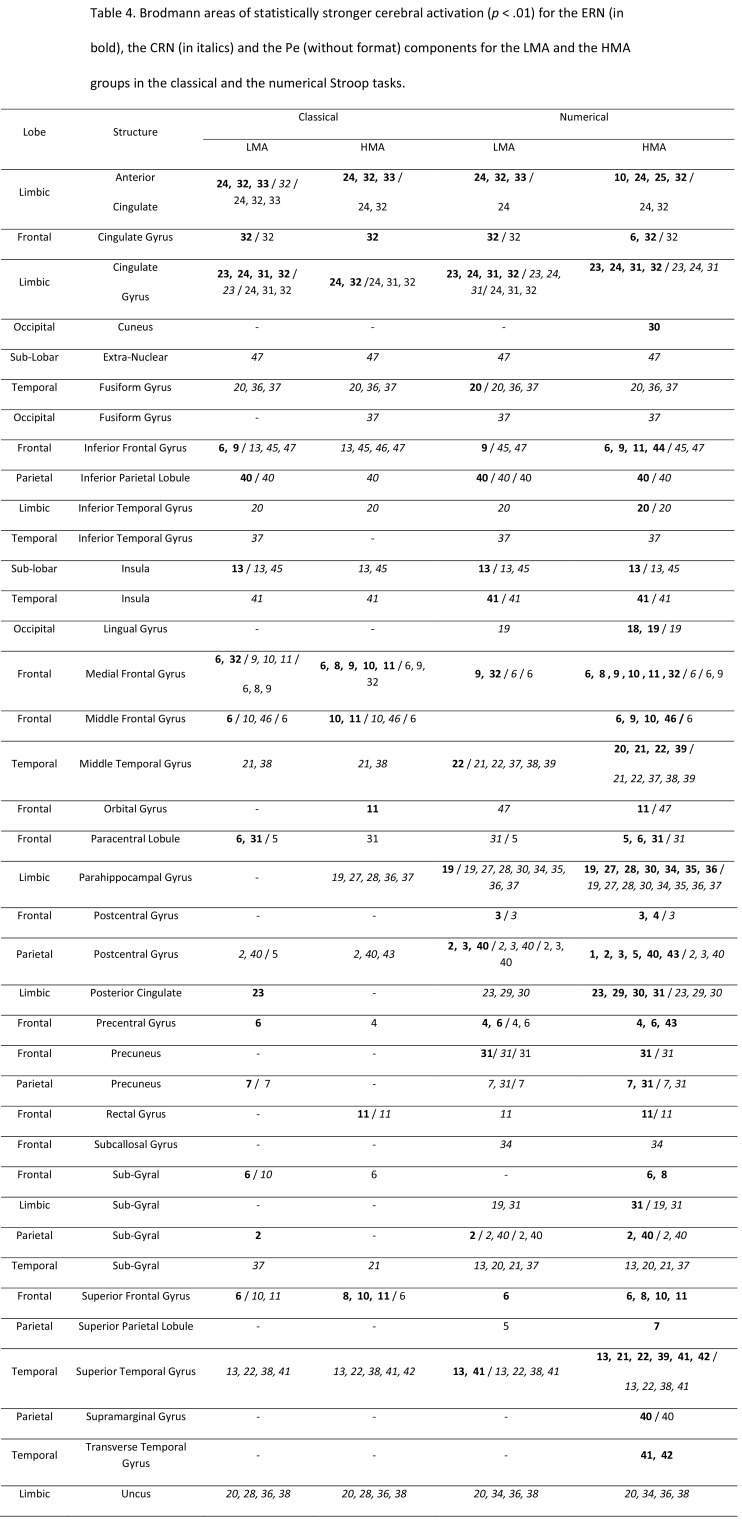


In "The numerical Stroop task" section of the Materials, the numbers are not correctly formatted. The sentence should read “in the congruent condition, the number of larger numerical magnitude was also larger in physical size (e.g. 8 9), in the incongruent condition, the number of larger numerical magnitude was smaller in physical size (e.g. 8 9) and in the neutral condition the numbers only differed in numerical magnitude, but not in physical size (e.g. 8 9)”, with the first 9 being larger, the second 8 being larger, and the third set of numbers being the same size.

In the "Source Localization" section of the Results, under the subheading "For the ERN", "(in red)" should be replaced by "(in bold)". Under the subheading "For the CRN", "(in blue)" should be replaced by "(in italics)". Lastly, under the subheading "For the Pe component", "(in green)" should be replaced by "(without format)."

